# Using Mobile Video-Teleconferencing to Deliver Secondary Stroke Prevention Interventions: A Pilot Study

**DOI:** 10.1089/tmr.2022.0026

**Published:** 2022-09-20

**Authors:** Jane A. Anderson, Barbara Kimmel, Shubhada Sansgiry, Chethan P. Venkatasubba Rao, Anette P. Ovalle, Colleen A. Cerra-Stewart, Thomas A. Kent

**Affiliations:** ^1^ Department of Medicine, Houston VA HSR&D Center for Innovations in Quality, Effectiveness and Safety, Michael E. DeBakey Veterans Affairs Medical Center, Houston, Texas, USA.; ^2^ Department of Neurology, and Baylor College of Medicine, Houston, Texas, USA.; ^3^ Department of Medicine, Baylor College of Medicine, Houston, Texas, USA.; ^4^ Department of Medicine, South Central Mental Illness Research, Education and Clinical Center, Houston, Texas, USA.; ^5^ Department of Neurosurgery, Baylor College of Medicine, Houston, Texas, USA.; ^6^ Center for Space Medicine, Baylor College of Medicine, Houston, Texas, USA.; ^7^ Center for Genomic and Precision Medicine, Institute of Bioscience and Technology Texas A&M Health Science Center, Texas, USA.; ^8^ Houston Methodist Hospital, Neurological Institute, Houston, Texas, USA.; ^9^ Department of Chemistry, Rice University, Houston, Texas, USA.

**Keywords:** m-health, self-management, secondary stroke prevention, stroke survivors, telehealth, underserved

## Abstract

**Objectives:**

Patient self-management support (SMS) interventions help stroke survivors control stroke risk factors and assist with secondary prevention. We examined utility and preliminary effectiveness of mobile video-teleconferencing (VT) to deliver SMS to stroke survivors in rural and low-income urban Texas communities.

**Methods:**

We applied a within-subjects design to assess improvement in self-management behaviors and stroke risk factors among stroke survivors receiving SMS intervention through mobile VT. Adults with stroke and two or more uncontrolled stroke risk factors were eligible. The SMS program, Video-teleconference-Self-management TO Prevent stroke (V-STOP) was delivered over 6 weeks by trained health coaches through VT. We applied Generalized Estimating Equations with site and time in intervention as covariates to evaluate psychological, social, physiological outcomes, self-management behaviors, and quality of life.

**Results:**

Mean age of 106 participants was 59.3 (±10.9); most were White, Hispanic men, living with someone, with low income. Approximately 69% completed all measures at 6 weeks. Median number of sessions attended was 5 (interquartile range 3) potentially avoiding 210 km of travel per person. Satisfaction with V-STOP and VT delivery was high, at (4.8 [±0.5]) and (4.7 [±0.5]), respectively. Stroke knowledge was improved from 8.8 (±1.0) at baseline to 9.6 (±0.7) at 12 weeks, (*p* < 0.0001). Improvements were observed in self-efficacy, exercise behaviors, depression and anxiety, disability, and quality of life.

**Conclusion:**

Implementation of SMS is feasible and shows good utility and preliminary effectiveness of using mobile VT to provide stroke follow-up care to stroke survivors. Participants improved self-management behaviors and stroke risk factors.

## Introduction

In the United States, ∼800,000 people have a stroke each year, resulting in tremendous personal and financial burden. For decades stroke has remained the leading cause of long-term disability worldwide.^1,2^ Unfortunately, 30% of strokes that occur each year are second strokes, which have poorer outcomes and are costlier than first strokes. Most strokes are preventable through management of controllable vascular risk factors.^3–5^ Prevention of second stroke and other vascular events requires secondary prevention interventions that target stroke etiology and incorporate individualized risk-factor management plans. Self-management support (SMS) interventions have been developed to help stroke survivors meet the difficulties and challenges of stroke recovery and secondary prevention.^6–10^ However, evidence-based delivery methods for SMS within the poststroke care continuum and under less structured clinical conditions remain undetermined. Some previous studies indirectly address the principal questions being asked in this study.^11–13^

We believe that no studies have been conducted to address the best delivery method for SMS using programs like Video-teleconferencing Self-management TO Prevent stroke (V-STOP) using mobile video teleconferencing (VT), as the V-STOP program was designed for secondary stroke prevention specifically related to the self-management of controllable stroke risk factors.

However, recently a few more studies have been published that synthesize the evidence of the effectiveness of SMS in improving health-related outcomes for stroke survivors using telehealth. These reviews discovered a positive effect on various self-management outcomes for poststroke patients. However, the authors recognized that the tailored interventions with well-defined content and mode of telehealth delivery with targeted outcomes and quality measures should further be explored.^14–16^

Therefore, with recent exponential growth in telehealth, effective delivery of SMS using mobile VT applications needs to be examined.^17–21^ Mobile VT has great potential to improve access to secondary stroke prevention and SMS for stroke survivors living in rural and low-income urban communities.^22^

## Methods

Multiple methods were applied to establish utility and preliminary effectiveness of using mobile VT to provide stroke follow-up care and SMS to stroke survivors living in rural and low-income urban communities in Texas.^23^ The investigators applied a within-subjects pilot design to assess improvement in self-management behaviors and stroke risk factors among stroke survivors receiving a stroke SMS intervention while at home through a VT application. We used a VT application that was accessible on mobile devices and personal computers. However, all participants in the study used a mobile device such as a smartphone or tablet computer to participate in the intervention.

### Setting and sample selection

The study was implemented within the Lone Star Stroke Consortium (LSSC; https://lonestarstroke.com), a research network of academic medical centers (hub sites) linked to affiliate community hospitals and clinics (spokesites) across Texas. Institutional Review Board approval was obtained from the LSSC academic hub site and coordinating center. Convenience sampling was applied, using an electronic medical record (EMR) algorithm in five LSSC affiliate spoke sites that serve higher numbers of stroke survivors from rural and low-income urban communities.

### Criteria for participation

Patients >18 years of age receiving care in an LSSC facility were screened for participation, based on the following criteria: a history of ischemic stroke within 12 months and two or more uncontrolled stroke risk factors; the ability to read and speak English or Spanish; and access to a smartphone, tablet, or computer with capability to load a mobile VT application. Patients with severe cognitive deficits, aphasia, medical record documentation of being medically or mentally unstable, or inability to follow-up (homelessness or incarceration) were excluded.

### Participant enrollment

Screening and recruitment were conducted between March 2017 and June 2019. A sampling pool of 1900 patients aged 18 years or older, with a diagnosis of ischemic stroke within the past 12 months, was identified, using an EMR algorithm. Prescreening through EMR chart review was completed on all patients. A total of 715 patients were excluded from the sampling pool, leaving 1185 patients identified for screening. Initial contact to introduce the study was established during hospital admission or during a follow-up clinic visit. Patients indicating interest in the study were contacted through telephone to complete the screening questionnaire.

**FIG. 1.**
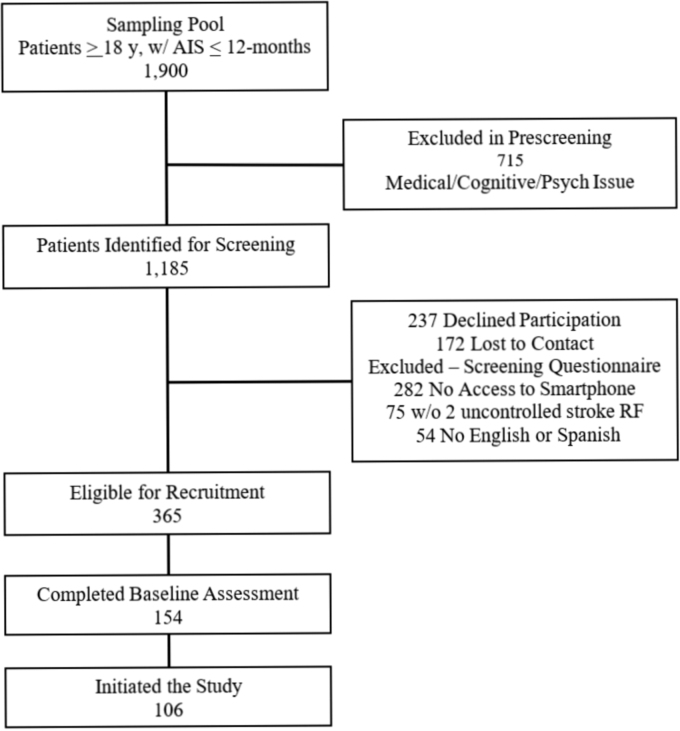
Organizational flowchart showing study recruitment. AIS, acute ischemic stroke; RF, risk factors.

Among the 1185 patients identified for screening, 365 (31%) patients were identified for recruitment. Of those, 193 (53%) signed informed consent and enrolled in the study. Of patients enrolled, 154 (78%) completed the baseline assessment and 106 (55%) initiated the study by completing at least one V-STOP intervention session through mobile VT (Fig. 1).

### Study procedures

The SMS intervention was V-STOP—a stroke SMS program developed by the investigators and adapted for telehealth delivery in the Veterans Health Administration.^6–8^ The intervention was delivered using a centralized telehealth delivery model. Five trained health coaches at the LSSC hub site used a point-to-point mobile VT application to deliver V-STOP to stroke survivors at home through their personal smartphone, tablet, or computer. All coaches had graduate degrees that focused on health prevention and promotion, such as public health and nursing. Each of the three coaches carried out exactly 19 group sessions. Of the two coaches conducting individual sessions, Coach 1 carried out 38, and Coach 2, 19 sessions.

The V-STOP intervention is a 6-week SMS program that consists of three group education sessions and three individualized coaching sessions. Group sessions were delivered simultaneously over the 6 consecutive weeks to up to 10 participants per session, who were invited to attend by clicking on a mobile VT meeting link sent through e-mail. Sessions were for 90 min and focused on development of self-management skills and strategies for addressing the specific and unique needs of stroke survivors. All sessions were provided using a scripted format to ensure implementation fidelity.

Individualized coaching sessions were delivered one-on-one to each participant. A trained health coach met individually with each participant, using a mobile VT meeting link sent through e-mail. Each coaching session was 30 min. Participants were coached in identification and management of their personal stroke risk-factor profile and guided in developing personalized self-management goals to reduce stroke risk. A secondary stroke-prevention and self-management protocol was used to guide each coaching session.

### Data collection

Multiple repeated measures were collected at baseline, 6, 12, and 18 weeks to determine the utility and preliminary effectiveness of using mobile VT for secondary stroke prevention and SMS. Study data were collected and managed using Research Electronic Data Capture (REDCap; Vanderbilt University, Nashville, TN, USA), hosted by the LSSC hub site. REDCap is a secure web-based software platform designed to support data capture for research studies.

### Measures

A baseline questionnaire was used to collect participants' sociodemographic characteristics and personal health history. Utility was measured as access and acceptability of the V-STOP intervention. Access was determined as intervention attendance and completion rates. Acceptance was assessed using the V-STOP satisfaction survey and the Telemedicine Satisfaction Questionnaire. Multiple validated self-reported measures were used to determine preliminary effectiveness of the V-STOP intervention on participants' self-management behaviors, stroke risk knowledge, psychological and physiological outcomes, and quality of life^24–26^ (Table 1).

**Table 1.** Assessment Measures

Outcome	Assessment measure
Self-management skills	SES^27^
	GAM-s^28^
	EBS^27^
Stroke risk knowledge	Stroke risk questionnaire^29^
Psychosocial health outcomes	General Anxiety Disorders (GAD-7)^30^
	Depression scale (PHQ-8)^31^
Physiological health outcomes	BP
	Disability HAQ-8 scale^32^
	BMI
QOL	SF-12^33^

BMI, body mass index; BP, blood pressure; EBS, Exercise Behavior Scale; GAD-7, 7-item Generalized Anxiety Disorder; GAM-s, goal attainment measure-stroke; HAQ-8, Health Assessment Questionnaire for Depression; PHQ-8, 8-item Patient Health Questionnaire for depression; QOL, quality of life; SES, Self-Efficacy Scale; SF-12, 12-item Short Form Survey.

### Analysis plan

Participants' sociodemographic characteristics, access, and acceptability measures were analyzed descriptively. Stroke risk knowledge was assessed with paired *t*-test. Means and medians were calculated for continuous variables and frequency for categorical variables. Longitudinal analysis was conducted for SMS effectiveness, using the Generalized Estimating Equations with site and time in intervention as covariates, using SAS^®^ 9.4 software (SAS Institute, Cary, NC, USA). Outcomes evaluated were psychological (anxiety and depression), social (community integration), physiological (body mass index [BMI], blood pressure [BP], and disability), self-management behaviors (exercise behavior, self-efficacy, and goal attainment), and health-related quality of life.

## Results

### Sociodemographic characteristics

V-STOP was successfully delivered using mobile VT to 106 participants for 2 years. Mean age was 59.3 (±10.9), most were White, Hispanic, men, not living alone, and married, with low annual income, and lacking insurance. Demographic characteristics are presented in Table 2.

**Table 2.** Participant Characteristics at Baseline (*N* = 106)

Characteristics	Frequency (%) ***n*** = 106
Age years (mean ± SD)	59.3 ± 10.9
Gender	
Male	58 (54.7)
Female	48 (45.3)
Race	
White	87 (82.1)
Black/African American	18 (17.0)
Native American/Alaska Native	1 (0.9)
Ethnicity	
Hispanic/Latino	66 (62.3)
Non-Hispanic/Latino	40 (37.7)
Education	
Less than high school	23 (21.7)
High school	41 (38.7)
Some college	26 (24.5)
College graduate or higher	16 (15.1)
Living status	
Alone	15 (14.2)
With someone	91 (85.9)
Marital status	
Single	50 (47.2)
Married	56 (52.8)
Employment	
Not employed	26 (24.5)
Employed/self employed	29 (27.4)
Disabled	23 (21.7)
Retired	27 (25.5)
Unknown/missing	1 (0.9)
Annual income	
<$25,000	62 (58.5)
$25,000–$49,999	25 (23.6)
$50,000–$74,999	11 (10.4)
$75,000–$99,999	3 (2.8)
Unknown	5 (4.7)
Insurance coverage	
Yes	63 (59.4)
No	43 (40.6)
Rurality	
Urban	69 (65.1)
Rural	30 (28.3)
Missing/unknown	7 (6.6)
Comorbidities	
Arthritis	26 (24.8)
Asthma	16 (15.2)
Atrial fibrillation	11 (10.5)
Heart failure	28 (26.7)
Hypertension	92 (87.6)
Diabetes	63 (60.0)
Depression	22 (21.0)
Baseline PHQ-8 ≥ 10	32 (30.5)
Baseline GAD-7 ≥ 10	32 (30.5)

SD, standard deviation.

### Utility: access and acceptability of mobile VT delivery

Approximately 54% of baseline participants completed all study questionnaire measures at 18 weeks, with 60% completing all measures at 12 weeks, and 69% completing all measures at 6 weeks. Of the study initiators (*n* = 106), 52 (49.1%) completed all six V-STOP intervention sessions; and 17 (16.0%) completed five sessions. Median (interquartile range [IQR]) number of sessions attended were 5 (IQR 3). Delivery of V-STOP using mobile VT eliminated 131 miles of travel per person over the 6-week intervention. Overall satisfaction with V-STOP and VT delivery was high at (4.8 [±0.5]) and (4.7 [±0.5]), respectively. Participants' stroke knowledge was improved from 8.8 (±1.0) at baseline to 9.6 (±0.7) at 12 weeks, *p*-value <0.0001. Longitudinal analyses data for study outcomes are shown in Table 3.

**Table 3.** Longitudinal Analyses for Study Outcomes

Domain	Outcome	Time point (weeks)	β estimate	95% confidence limits	***p***
	**Psychosocial outcomes**				
Psychological	Depression (PHQ-8)	BL (ref)			
		6	0.6386	0.5316–0.7670	<0.0001^*^
		12	0.6789	0.5289–0.8715	0.0024^*^
		18	0.6612	0.4973–0.8791	0.0044^*^
Psychological	Anxiety (GAD-7)	BL (ref)			
		6	0.6639	0.5483–0.8040	<0.0001^*^
		12	0.6067	0.4784–0.7693	<0.0001^*^
		18	0.6561	0.5096–0.8447	0.0011^*^
Social	Community integration (CIQ)	BL (ref)			
		6	1.0627	0.9803–1.1522	0.0285^*^
		12	1.0118	0.9407–1.0883	0.7525
		18	1.0810	1.0082–1.1590	0.1399
	**Physiological outcomes**				
Physiological	BMI	BL (ref)			
		6	1.0254	0.9455–1.1119	0.5450
		12	1.0071	0.8978–1.1297	0.9036
		18	0.9412	0.8823–1.0040	0.0661
Physiological	BP (diastolic)	BL (ref)			
		6	1.0581	1.0175–1.1003	0.0047^*^
		12	0.9880	0.9418–1.0364	0.6206
		18	1.0068	0.9557–1.0607	0.7978
Physiological	BP (systolic)	BL (ref)			
		6	1.0123	0.9650–1.0618	0.6169
		12	0.9883	0.9451–1.0335	0.6069
		18	0.9783	0.9265–1.0331	0.4306
Physiological	Disability and function (HAQ disability)	BL (ref)			
		12	0.8311	0.6413–1.0772	0.1621
		18	0.6492	0.5112–0.8244	0.0004^*^
	**Self-management**				
Self-management	Exercise behavior (time in minutes)	BL (ref)			
		12	1.4881	1.1743–1.8856	0.0010^*^
		18	1.7670	1.4248–2.1915	<0.0001^*^
Self-management	Self-efficacy for managing chronic disease (SE)	BL (ref)			
		12	1.1279	1.0547–1.2061	0.0004^*^
		18	1.1779	1.1076–1.2527	<0.0001^*^
Self-management	GAM-s	3 (ref)			
		6	0.9596	0.8924–1.0317	0.2645
		12	0.8878	0.8222–0.9587	0.0024^*^
		18	0.9154	0.8444–0.9925	0.0321^*^
	**QOL**				
QOL	PCS QOL (SF-12)	BL (ref)			
		12	1.0296	0.9689–1.0940	0.3469
		18	1.1114	1.0450–1.1820	0.0008^*^
QOL	MCS QOL (SF-12)	BL (ref)			
		12	1.1017	1.0441–1.1625	0.0004^*^
		18	1.1075	1.0550–1.1625	<0.0001^*^

*Note:* All longitudinal models are adjusted for study site and time in intervention.

^*^ Represents significant *p* values.

BL, baseline; CIQ, Community Integration; MCS, mental component score; PCS, physical component score.

### Self-management behaviors

#### Self-efficacy

Self-efficacy for managing chronic disease was measured using a 10-point level of confidence ranging from not at all, 0, to totally confident, with higher scores indicating higher levels of confidence.^27^ Mean (±standard deviation) and median (IQR) for self-efficacy (SE) was 7.0 (±2.0) and 7 (IQR 2.5) at baseline, 7.7 (±1.8) and (IQR 3.2) at 12 weeks, and 8.1 (±1.7) and 8.2 (IQR 2.8) at 18 weeks. SE increased from baseline to 12 weeks (β = 1.1, 95% confidence interval [CI] 1.05 to 1.21) and from baseline to 18 weeks (β = 1.2, 95% CI 1.11 to 1.25).

#### Exercise behavior

Exercise behavior was measured as total time (in minutes) spent exercising per week. Mean number of minutes spent exercising was 117.5 (±128.0) at baseline, 191.5 (±128.1) at 12 weeks, and 207.4 (±144.7) at 18 weeks. Exercise behavior improved with more time spent exercising from baseline to 12 weeks (β = 1.49, 95% CI 1.17 to 1.19) and baseline to 18 weeks (β = 1.77, 95% CI 1.43 to 2.19). There was no change from 12 to 18 weeks (*p* = 0.07).

#### Goal attainment measure

Goal attainment was measured beginning at 3 weeks as ability to complete assigned task, with higher score indicating better ability. Total participants at 3 and 12 weeks was *n* = 60 (100%) but dropped to *n* = 58 (96.8%) at 18 weeks. Mean goal attainment was 7.0 (±1.9) at 3 weeks, 7.0 (±2.0) at 4 weeks, 7.4 (±1.4) at 5 weeks, 7.1 (±1.6) at 6 weeks, 6.4 (±2.0) at 12 weeks, and 6.2 (±2.4) at 18 weeks. Goal attainment was lower at 12 weeks (β = 0.89, 95% CI 0.82 to 0.96), as well as at 18 weeks when compared with 3 weeks (β = 0.91, 95% CI 0.84 to 0.99) but did not change at 4 or 5 weeks when compared with score at 3 weeks (*p* > 0.05).

### Psychological health outcomes

#### Depression

Depression was measured by the 8-item Patient Health Questionnaire for depression (PHQ-8), with higher score indicating higher depression. Mean PHQ-8 score at baseline was 6.5 (±5.9), 6-week mean score was down to 3.9 (±4.1), 12-week score was 4.1 (±4.5), and 18-week score was 3.5 (±4.1). Depression scores improved (*p* < 0.05) from baseline to 6 weeks (β = 0.62, 95% CI 0.51 to 0.75), baseline to 12 weeks (β = 0.66, 95% CI −0.52 to 0.85), and baseline to 18 weeks (β = 0.64, 95% CI 0.49 to 0.84). The improvement at 6 weeks was maintained for 12 and 18 weeks.

#### Anxiety

Anxiety was measured using the 7-item Generalized Anxiety Disorder (GAD-7) assessment tool, with higher score indicating higher anxiety. Mean GAD-7 score at baseline was 6.4 (±6.9), 6-week mean score was 4.1 (±4.6), 12-week score was 3.4 (±4.7), and 18-week score was 3.3 (±4.7). Anxiety scores improved (*p* < 0.05) from baseline to 6 weeks (β = 0.66, 95% CI 0.54 to 0.80), baseline to 12 weeks (β = 0.61, 95% CI −0.48 to 0.77), and baseline to 18 weeks (β = 0.66, 95% CI 0.51 to 0.85). The improvement at 6 weeks was maintained for 12 and 18 weeks.

### Physiological health outcomes

#### Body mass index

Attrition from baseline for physiological health outcomes was very high in this study sample. BMI assessment (self-reported) was 15% at 6 weeks, 12% at 12 weeks, and 14% at 18 weeks. BMI mean was 32.7 (±8.0) at baseline, 34.8 (±9.9) at 6 weeks, 35.5 (±9.0) at 12 weeks, and 30.8 (±7.4) at 18 weeks. Longitudinal analysis did not indicate any change in BMI.

#### Blood pressure

Attrition for self-reported BP measurement was also very high, with only 44% completing BP measure at 6 weeks, 12% at 12 weeks, and 22.6% at 18 weeks. Mean systolic and diastolic BP at baseline was 134.2 (±23.9) and 77.3 (±11.1), respectively; at 6 weeks was 134.5 (±22.3) and 81.8 (±10.5), respectively; at 12 weeks was 130.7 (±16.2) and 76 (±11.7), respectively; and at 18 weeks was 129 (±16.8), and 77.3 (±9.4), respectively.

Diastolic BP increased from baseline to 6 weeks (β = 1.1, 95% CI 1.02 to 1.10) but did not indicate any change between other time points (*p* > 0.05). Systolic BP also did not show any change between any two time points (*p* > 0.05).

#### Disability

Disability was measured using the Stanford Health Assessment Questionnaire, with higher score indicating higher perceived difficulty with daily activities. Mean score was 0.42 (±0.55) at baseline, 0.34 (±0.48) at 12 weeks, and 0.20 (±0.33) at 18 weeks. Perceived difficulty was reduced from baseline to 18 weeks (β = 0.65, 95% CI 0.51 to 0.82), but no change was observed from baseline to 12 weeks (*p* = 0.16).

### Quality of life

Quality of life was measured using the 12-item Short Form Survey questionnaire with two component scores, physical and mental. Higher score indicates higher quality of life. Mean physical component score (PCS) and mental component scores (MCS) were, respectively, 41.8 (±10.6) and 47.5 (±11.0) at baseline, 42.7 (±10.0) and 51.7 (±11.4) at 12 weeks, and 46.8 (±8.6) and 52.3 (±8.9) at 18 weeks. PCS improved from baseline to 18 weeks (β = 1.1, 95% CI 1.05 to 1.18). However, no change was observed from baseline to 12 weeks (*p* = 0.35). The improvement in PCS was seen from 12 to 18 weeks (β = 1.1, 95% CI 1.03 to 1.14). Similar to PCS, the MCS improved from baseline to 18 weeks (β = 1.1, 95% CI 1.06 to 1.16). Improvement in MCS was also seen from baseline to 12 weeks (β = 1.1, 95% CI 1.04 to 1.16).

Longitudinal analyses for study outcomes are presented in Table 3.

## Discussion

Expansion of telehealth services may be an important strategy to mitigate challenging social determinants of health and health vulnerability.^17,34,35^ In this pilot study we examined the utility and effectiveness of using a mobile VT application to deliver secondary stroke prevention and SMS to a sample of stroke survivors challenged by low income and other social determinants of health. It has already been mentioned that health interventions delivered through mobile VT have strong empirical support from research studies. However, their implementation and effectiveness under less structured clinical conditions have not been well demonstrated.

Results indicate mobile VT is feasible for delivery of secondary stroke prevention and SMS among stroke survivors with health vulnerability. Mobile VT delivery showed good utility as an alternative to traditional in-person clinic follow-up. Attendance and completion rates for V-STOP were comparable with traditional in-person SMS programs.^36,37^ Moreover, high satisfaction scores for the SMS program and mobile VT delivery showed VT to be highly acceptable among participants. Finally, access to SMS and secondary stroke prevention was improved by eliminating 131 miles of in-person travel for participants over the 6-week intervention, thus supporting telehealth modalities as a strategy to improve access to stroke follow-up care.

Study findings indicate SMS delivered using mobile VT is effective for managing depression and anxiety and supports community integration after stroke. During the study, participants were encouraged to set risk-reduction goals using goal setting and action planning methodology.^38^ Our results parallel existing evidence from the systematic review reporting positive effects of the telemedicine SMS interventions on all self-efficacy outcomes.^16^ However, it is important to point out that the telehealth delivery type most used in selected studies was messaging.

Participants also demonstrated significant improvement in stroke risk knowledge and confidence for managing stroke risk factors. Upon completion of V-STOP, participants showed significant improvement in perceived disability and overall quality of life.

More research is needed to determine how best to incorporate telehealth modalities into routine clinical practice for delivery of stroke follow-up care.^16^ Our research raises several important questions that require further investigation. We experienced challenges with recruitment and retention. Many patients were excluded due to being medically unstable at the time of recruitment. A primary reason may be related to having a sampling pool that included currently hospitalized stroke survivors. Participants' attrition and visit compliance were an issue.

Similar problems were reported by Kendall et al^39^ and Cadilhack et al.^40^ In addition, self-reported data contributed to a response bias, hence creating problems with accurate measures to model health outcomes. To control response bias, part of the group educational sessions was devoted to educating participants in self-reported data collection. We provided trainings on the correct way to measure BP and factors that affect BP readings. For additional information we provided relevant links to the American Stroke Association and Centers for Disease Control websites.

The organizational structure in which the program was delivered, staff turnover, and a lack of deeper understanding of the individual health challenges of medically underserved stroke survivors need to be addressed in future studies.

Overall, this pilot study was successful in establishing the feasibility, utility, and preliminary effectiveness of using mobile VT to provide stroke follow-up care and SMS to stroke survivors in medically underserved communities in Texas. The study showed improvement in self-efficacy and exercise behavior receiving a stroke SMS intervention while at home through mobile VT.

Although this is encouraging, this observational pilot study has limited generalizability, as there was no randomization; and we targeted a small sample of medically underserved stroke survivors in Texas. This was intentional as a first step in evaluating mobile VT as a strategy to provide secondary stroke prevention and SMS to health-vulnerable communities in Texas. A future study with a large sample size and inclusion of a comparison group is planned.

## Abbreviations Used

BMI: body mass index
BP: blood pressure
CI: confidence interval
EBS: Exercise Behavior Scale
EMR: electronic medical record
GAD-7: 7-item Generalized Anxiety Disorder
GAM-s: goal attainment measure-stroke
IQR: interquartile range
LSSC: Lone Star Stroke Consortium
MCS: mental component score
PCS: physical component score
PHQ-8: 8-item Patient Health Questionnaire for depression
QOL: quality of life
REDCap: Research Electronic Data Capture
SD: standard deviation
SES: Self-Efficacy Scale
SF-12: 12-item Short Form Survey
SMS: self-management support
V-STOP: Video-teleconferencing Self-management TO Prevent stroke
VT: video teleconferencing
